# Additive value of blood neutrophil gelatinase-associated lipocalin to clinical judgement in acute kidney injury diagnosis and mortality prediction in patients hospitalized from the emergency department

**DOI:** 10.1186/cc12510

**Published:** 2013-02-12

**Authors:** Salvatore Di Somma, Laura Magrini, Benedetta De Berardinis, Rossella Marino, Enrico Ferri, Paolo Moscatelli, Paola Ballarino, Giuseppe Carpinteri, Paola Noto, Biancamaria Gliozzo, Lorenzo Paladino, Enrico Di Stasio

**Affiliations:** 1 Department of Emergency Medicine, Medical-Surgery Sciences and Translational Medicine, S. Andrea Hospital, 'Sapienza' University, via di Grottarossa 1035-1039, Rome 00189, Italy; 2 Department of Emergency Medicine, IRCC AOU S. Martino - IST University Hospital, Largo Rosanna Benzi 10, Genoa 16132, Italy; 3 Department of Emergency Medicine, Vittorio Emanuele University Hospital, via S. Sofia 78, Catania, 95123, Italy; 4 Department of Emergency Medicine, SUNY Downstate - Kings County Hospital Medical Center, 450 Clarkson Ave, New York, NY 11203, USA; 5 Institute of Biochemistry and Clinical Biochemistry, Catholic University of Sacred Heart, Largo Agostino Gemelli 8, Rome 00168, Italy

## Abstract

**Introduction:**

Acute kidney injury (AKI) is a common complication among hospitalized patients. The aim of this study was to evaluate the utility of blood neutrophil gelatinase-associated lipocalin (NGAL) assessment as an aid in the early risk evaluation for AKI development in admitted patients.

**Methods:**

This is a multicenter Italian prospective emergency department (ED) cohort study in which we enrolled 665 patients admitted to hospital from the ED.

**Results:**

Blood NGAL and serum creatinine (sCr) were determined at ED presentation (T0), and at: 6 (T6), 12 (T12), 24 (T24) and 72 (T72) hours after hospitalization. A preliminary assessment of AKI by the treating ED physician occurred in 218 out of 665 patients (33%), while RIFLE AKI by expert nephrologists was confirmed in 49 out of 665 patients (7%). The ED physician's initial judgement lacked sensitivity and specificity, overpredicting the diagnosis of AKI in 27% of the cohort, while missing 20% of those with AKI as a final diagnosis.

The area under the receiver operating characteristic curve (AUC), obtained at T0, for blood NGAL alone in the AKI group was 0.80. When NGAL at T0 was added to the ED physician's initial clinical judgment the AUC was increased to 0.90, significantly greater when compared to the AUC of the T0 estimated glomerular filtration rate (eGFR) obtained either by modification of diet in renal disease (MDRD) equation (0.78) or Cockroft-Gault formula (0.78) (*P *= 0.022 and *P *= 0.020 respectively). The model obtained by combining NGAL with the ED physician's initial clinical judgement compared to the model combining sCr with the ED physician's initial clinical judgement, resulted in a net reclassification index of 32.4 percentage points. Serial assessment of T0 and T6 hours NGAL provided a high negative predictive value (NPV) (98%) in ruling out the diagnosis of AKI within 6 hours of patients' ED arrival. NGAL (T0) showed the strongest predictive value for in-hospital patient's mortality at a cutoff of 400 ng/ml.

**Conclusions:**

Our study demonstrated that assessment of a patient's initial blood NGAL when admitted to hospital from the ED improved the initial clinical diagnosis of AKI and predicted in-hospital mortality. Blood NGAL assessment coupled with the ED physician's clinical judgment may prove useful in deciding the appropriate strategies for patients at risk for the development of AKI.

See related commentary by Legrand *et al., *http://ccforum.com/content/17/2/132

## Introduction

Acute kidney injury (AKI), defined as an abrupt decrease in renal function over a period of hours to days, is a common complication among hospitalized patients. Its incidence has been increasing in recent years [1-3], and is reported to be very high (11%) in the emergency department (ED) setting [4 -5]. Since clinical signs and symptoms of acute renal damage are not specific [3,6,7] it is difficult to promptly distinguish AKI at the time of ED presentation. Currently the diagnosis of AKI requires serial assessment of laboratory tests over a period of several days, and is based mainly on serum creatinine (sCr) as supported by Risk, Injury, Failure, Loss, and End-Stage Kidney Disease (RIFLE) criteria, Acute Kidney Injury Network (AKIN) criteria and the recent Kidney Disease: Improving Global Outcomes (KDIGO) practice guidelines for AKI [8-10]. This need for repeated sCr evaluations and monitoring of urinary output for several days after admission could therefore result in a delay in appropriate therapy [3,8-10]. Moreover the application of the RIFLE criteria to patients presenting to ED is quite challenging since decrease in urine output is not quantified, and a prehospital stable baseline sCr is in most of the cases not available.

As a consequence, the use of biomarkers of acute kidney damage could be of great utility in the ED in order to distinguish AKI from volume responsive renal dysfunction, chronic kidney disease (CKD) or normal renal function [4,5,11]. Furthermore these biomarkers could contribute to the diagnosis of AKI by identifying a subgroup with 'subclinical AKI' where there may be injury even in the absence of a sCr increase [12,13], thus leading to an earlier risk stratification of patients with prompt and specific treatment strategies.

Among damage biomarkers of AKI, the largest body of evidence for the detection of AKI prior to sCr increase exists for both urine and plasma NGAL [4,5,13-21].

Nickolas *et al*., using a single urine NGAL measurement in the ED, demonstrated that this biomarker is superior to sCr in detecting AKI, and has a significant prognostic ability for these patients [4,5].

Although data exist on a specific acute disease ED population [22], so far no data have been reported on the utility of serial blood NGAL assessments in the diagnosis of AKI and in predicting in-hospital outcomes for general patient population presenting to the ED and requiring hospitalization.

The primary objective of this study was to evaluate the role of serial assessments of point-of-care (POCT) blood NGAL, compared to serial sCr assessments for the fast and accurate diagnosis of clinically adjudicated AKI in patients hospitalized from the ED for different acute diseases. Furthermore we evaluated if blood NGAL could have an additive value to the initial clinical judgement of the ED physician compared to the final expert adjudicated diagnosis of AKI in the acute care population. Secondary objectives included the evaluation of the utility of serial blood NGAL measurements and/or sCr as an aid in the detection of AKI defined by sCr changes, oliguria, and need for renal replacement therapy (RRT). Furthermore, we analyzed the utility of NGAL and sCr used in combination to predict the need for RRT and in-hospital mortality in the same cohort of patients.

## Materials and methods

This was a multicenter prospective clinical trial conducted in three EDs in Italy (Sant'Andrea Hospital in Rome - the coordinating centre, Vittorio Emanuele Hospital in Catania and San Martino Hospital in Genoa). The protocol was designed following the criteria of the Declaration of Helsinki and was approved by the ethical committee of Sant'Andrea Hospital (the coordinating center) and each participating hospital. Written informed consent was obtained by each patient prior to enrolment.

Inclusion criteria: we consecutively enrolled all patients in the ED who were designated to be admitted to the hospital for acute process in the period from November 2008 to April 2009.

Exclusion criteria: less than 18 years of age, patients with chronic renal insufficiency (sCr ≥ 3.0 mg/dL), on dialysis or RRT (either acute or chronic) or in imminent need of dialysis or RRT at enrolment; patients with urothelial malignancies, subjects not expected to be admitted and therefore unable to fulfil protocol requirements for blood collection at post admission time points; patients who had participated in an interventional clinical study within the previous 30 days, and patients unable to complete the informed consent of the study or to comply with study procedures.

### Data collection

Anamnestic data and demographic information were recorded. Instrumental diagnostic tests (EKG, chest X-ray, computed tomography (CT) scans, ultrasound assay and arterial blood gas analysis) were performed where necessary at the discretion of the treating physician and where not dictated by study protocol. Blood tests for hemochromocytometric examination, urea, creatinine, electrolytes, cardiac and hepatic enzymes, coagulation tests, and whole blood NGAL assay were performed for all patients. Each patient was treated on the basis of the formulated diagnosis and therapy was carefully recorded. The presence of concomitant clinical conditions such as CKD, diabetes and hypertension were evaluated on the basis of clinical history fulfilling the criteria of current guidelines [8,23,24].

**Figure 1:**
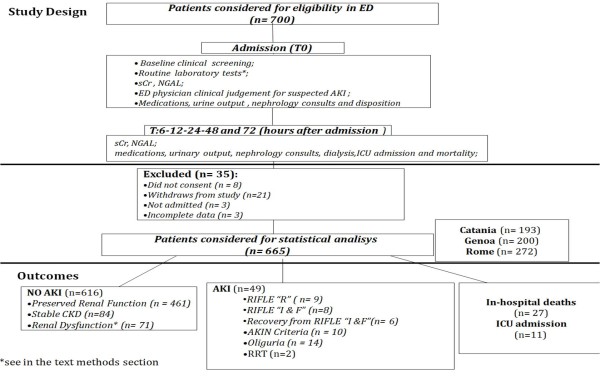
**Flow diagram of the study, patients' distribution and outcome**.

At the time of ED disposition of each enrolled patient, the ED treating physician was asked to note an initial clinical assessment of AKI versus NO AKI, and his level of confidence in that diagnosis as a percentage (0% to 100%) [25]. This assessment was following the initial examination and review of the patient's medical history, admission sCr levels, and demographic characteristics (Figure 1). There were no documented pre-study sCr levels available for any of the enrolled patients. Thus, the sCr level obtained at ED arrival was used as a baseline level for these patients.

Renal insults, the development of oliguria, the need for a nephrology consultation, initiation of dialysis or RRT (following KDIGO international guidelines)[10], Intensive Care Unit (ICU) admission and mortality were recorded through to discharge. Blood samples for NGAL and sCr were measured in all subjects at the following time points: T0, at 6 (T6) and 12 (T12) hours, and on days 1 (T24), 2 (T48) and 3 (T72) (Figure 1).

### Blood NGAL measurement

Blood samples were collected into tubes containing potassium ethylenediaminetetraacetic acid (EDTA) and NGAL was measured using the Triage NGAL assay (Alere Inc., San Diego, CA, USA). The Triage NGAL test is a point-of-care, fluorescence-based immunoassay for the rapid quantitative measurement of NGAL concentration in whole blood in the range 60 to 1300 ng/ml. The test procedure is performed by the addition of several drops of whole blood to the sample port on the test device. The sample reacts with fluorescent antibody conjugates in the reaction chamber, and flows down the reaction lane by capillary action. The conjugates are then captured on discrete solid-phase zones resulting in the binding of immunoassays specific for NGAL antigens. The concentration of the analyte is inversely correlated to the fluorescence detected. Therefore, the NGAL test is based on the amount of fluorescence the meter detects. The Triage platform is a POCT system designed for bedside use, capable of reporting a test result in 15 to 20 minutes and has been already used to measure NGAL [26]. The results of NGAL were blinded to the medical team during the study and did not impact the medical management of the subject.

### Blood NGAL cutoff 150 to 400 ng/ml

A cutoff of 150 ng/ml was selected in ruling out the diagnosis of AKI because it had previously been published as a high sensitivity threshold for AKI prediction [20,22]. It is also the published 95^th ^percentile of normal for the assay [22]. A cutoff of 400 ng/ml was selected as a high specificity threshold for AKI diagnosis based upon internal analysis that has been done on other sets of data [21].

### sCr and estimated glomerular filtration rate (eGFR) measurements

Creatinine was measured by an immunoenzymatic assay (Vitros Crea; Ortho Clinical Diagnostics, High Wycombe, UK). The normal range is 0.8 to 1.5 mg/dl. Estimated glomerular filtration rate (eGFR) was calculated by the modification of diet in renal disease (MDRD) equation based on four variables (age, sex, race and gender) recommended by the Kidney Disease Outcome Quality Initiative (KDOQI) guidelines [10,27]. eGFR obtained by Cockroft-Gault formula was also calculated at each considered time point in all patients [28].

### Kidney function - patients' classification

Based on patient case review, blinded to NGAL results, by two expert nephrologists at the coordinating center, each patient was assigned to one of the following diagnostic categories:

1. Preserved renal function: patients with eGFR ≥60 mL/min per 1.73 m^2 ^that did not meet the criteria of any of the other categories [8].

2. Stable chronic kidney disease (CKD): patients with a sustained elevation of sCr level indicative of a reduced eGFR of <60 mL/min per 1.73 m^2^, that did not elevate beyond the criteria for AKI and persisted for more than three months before hospitalization [8].

3. Renal dysfunction: patients with evidence of a new-onset increase in sCr level or decline in eGFR that exceeded the definition for AKI and either resolved within three days with treatment aimed at restoring perfusion (for example intravenous volume repletion or discontinuation of diuretics), or was accompanied by fractional excretion of sodium less than 1% at time of admission [5,13].

4. Acute kidney injury (AKI): as assigned by expert adjudication with consideration of the RIFLE criteria for urine output and eGFR changes during the patient's admission [8]. In particular a new-onset 1.5-fold increase in sCr level or 25% decreases in eGFR from baseline, or oliguria were considered. Secondary analysis using AKI endpoints based solely on sCr increase in 48 hours by AKIN criteria, by RIFLE criteria (I&F), including recovery from I and F and by oliguria were also performed [8,9]. Recovery from RIFLE 'I' and 'F' were defined by an in-hospital decrease of sCr value equal or greater than 100% and 200% respectively when using the lowest post peak sCr as a reference[8].

Stable CKD is not acute and renal dysfunction, as defined, is a reversible process and not reflective of intrinsic AKI, therefore, these two categories were included with normal preserved renal function as NO AKI.

### Statistical analysis

NGAL and sCr levels were presented as mean ± standard deviation (SD) for normally distributed data, and as median interquartile range (IQR) in case of abnormally distributed data.

The ED clinical confidence of AKI was correlated with the final diagnosis of AKI as defined above. The area under the curve (AUCs) of the receiver-operating characteristic (ROC), and odds ratios (OR) were calculated to quantify the accuracy of both NGAL and clinician judgment, individually and combined, in the prediction and assessment of AKI.

The utility of serial measurements of NGAL was also assessed for the same outcome. Because the aim of this study was the early recognition of AKI in the acute care setting, we specifically focused on the operating characteristics of NGAL in the first few hours: T0 and T6. The corresponding cutoffs and clinical performance parameters (sensitivity, specificity, positive predictive value (PPV), negative predictive value (NPV) and diagnostic accuracy) were evaluated. ROC curves using more than a single predictor (for example, NGAL and clinical judgment), are based on fitting a logistic regression model using the 'glm' package of R. The output of the model was then used for the ROC curve. Note that for a single predictor (for example, NGAL), the ROC curve based on an output of a logistic regression model is identical to the ROC curve based directly on the predictor, and any cut points will be the same when interpreted in terms of the predictor.

The correlation of baseline biomarkers, demographic variables, and clinical variables to clinical outcomes were assessed through box plots, ROCs, AUCs, and logistic regression. The 95% confidence intervals (95% CI) were calculated for AUCs. For each cutoff, the OR, sensitivity, specificity, PPV, NPV, and diagnostic accuracy were calculated along with and the 95% CI. Serial NGAL levels were assessed via box plots of the distribution at each blood draw time grouped by AKI versus NO AKI. Serial NGAL levels were assessed in absolute concentration, relative to baseline and relative to previous draw. The cutoff and estimated clinical sensitivity and specificity of the NGAL test to indicate the risk of these outcomes were calculated. The sensitivity and specificity of the physicians' clinical judgment for these outcomes was also calculated alone, and in combination with the Triage NGAL test results. Analysis of the potential reduction in clinical indecision provided by the NGAL test results was also performed.

The statistical significance was assessed by *t *test if data were normally distributed, otherwise by a nonparametric test, the Wilcoxon rank sum test as appropriate. The statistical significance of the association between dichotomous variables was assessed by the Fisher exact test and chi square test. A net reclassification index (NRI) analysis was used to assess improvement in the accuracy of the risk-prediction model for in-hospital mortality. The threshold for statistical significance was α <0.05. Revolution R Enterprise version 4.2 (Revolution Analytics, Palo Alto, CA, USA), SPSS version 14 (SPSS, Chicago, IL, USA) and Medcalc version12.1.4 (Medcalc Software, Mariakerke, Belgium) software were used.

## Results

### Baseline characteristics

Some 665 of the 700 patients enrolled (357M; 308F; mean age 74 ± 14.4 years) were included in the statistical analysis. Thirty-five patients were excluded: eight did not consent, twenty-one withdrew from the study, and six had incomplete data (Figure 1). Patients' characteristics are reported in Table 1. There was no significant difference in sex, age, body mass index (BMI) and blood pressure distribution within AKI and NO AKI groups of patients recorded at the time of admission. Chronic kidney disease and chronic heart failure related to cardiac valvular diseases were significantly more frequent in AKI patients when compared to NO AKI (respectively *P *< 0.03 and *P *< 0.04). The incidence of AKI was significantly higher in patients with in-hospital diagnosis of sepsis (*P *< 0.03) (Table 1). AKI was confirmed in 49 (7%) cases of 665 examined patients on the basis of expert nephrologists adjudication of cases: by RIFLE 'R' in 9, RIFLE 'I' and 'F' in 8, recovery from RIFLE 'I' and 'F' in 6 patients; by AKIN criteria in 10 patients; RRT in 2 patients and by oliguria in 14 patients (Figure 1). The ED physician's initial clinical judgment gave a presumptive diagnosis of AKI in 218 (32.7%) patients, but captured only 39 of those patients with a confirmed final adjudicated diagnosis of AKI, failing to identify the other 10.

**Table 1:** Patients' characteristics.

	TOTAL COHORT n (%)	PATIENTS WITH AKI n (%)	PATIENTS WITH NO AKI n (%)	*P *value
Total	665	49 (7.4%)	616 (92.6%)	*P *< 0.001

Men	358 (53.8%)	29 (8.1%)	329 (91.9%)	*P *< 0.001

Women	307 (46.2%)	20 (6.5%)	287 (93.5%)	*P *< 0.001

Mean age ±SD, y	74 ± 14.4	79.1 ± 10.7	73.7 ± 13.5	*P *= 0.42

Mean BMI ±SD(m^2^/h)	26.3 ± 4.7	25.8 ± 3.8	26.1 ± 4.3	*P *= 0.55

Mean systolic blood pressure ± SD *mmHg*	137 ± 58	128 ± 63	138 ± 55	*P *= 0.23

Mean diastolic blood pressure ±SD *mmHg*	77 ± 15	75 ± 18	77 ± 17	*P *= 0.44

	

**Past medical history**				

Coronary artery disease/chronic heart failure	271 (40.7%)	18/49 (36.7%)	253/616 (41.1%)	*P*= 0.50

Dyslipidemia	181 (27.2%)	5/49 (10.2%)	176/616 (28.5%)	*P *< 0.002

Cardiac arrhythmia	231 (34.7%)	13/49 (26.5%)	218/616 (35.3%)	*P *= 0.71

Chronic obstructive pulmonary disease	327 (49.1%)	17/49 (34.6%)	310/616 (50.3%)	*P *< 0.03

Hypertension	598 (89.9%)	35/49 (71.4%)	563/616 (91.3%)	*P *< 0.001

Chronic kidney disease	112 (16.8%)	14/49 (28.5%)	98/616 (15.9%)	*P *< 0.03

Cerebrovascular disease	129 (19.3%)	7/49 (14.2%)	122/616 (19.8%)	*P *= 0.41

Diabetes mellitus	294 (44.2%)	11/49 (22.4%)	283/616 (45.9%)	*P *< 0.001

Valvular disease/chronic heart failure	63 (9.4%)	10/49 (20.4%)	53/616 (8.6%)	*P *< 0.04

**Diagnosis at discharge**				

Sepsis	51 (7.6%)	11/49 (22.4%)	40/616 (6.4%)	*P *< 0.03

Local infections	121 (18.1%)	4/49 (8.1%)	117/616 (18.9%)	*P *< 0.04

Acute decompensated heart failure	108 (16.2%)	10/49 (20.4%)	98/616 (15.9%)	*P *= 0.51

Decompensated diabetes	10 (1.5%)	1/49 (2%)	9/616 (1.4%)	*P *= 0.82

Stroke	38 (5.7%)	4/49 (8.1%)	34/616(5.5%)	*P *= 0.61

Severe dehydration	9 (1.3%)	3/49 (6.5%)	6/616 (0.9%)	*P *< 0.08

Liver cirrhosis	11 (1.6%)	2/49 (4%)	9/616 (1.4%)	*P *= 0.43

AKI, acute kidney injury; BMI, body mass index; SD, standard deviation.

**Figure 2:**
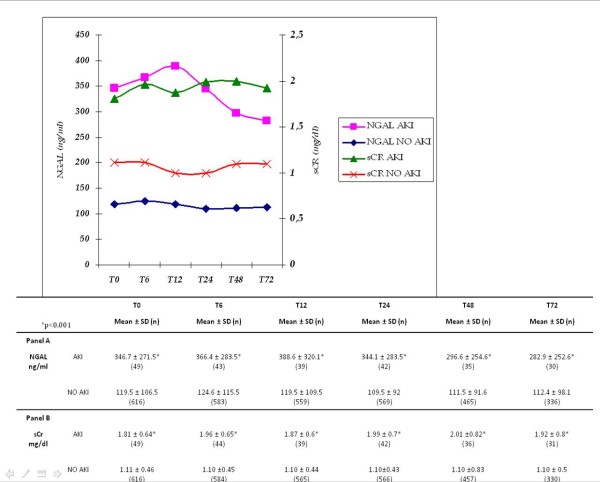
**Mean ± SD neutrophil gelatinase-associated lipocalin (NGAL) and serum creatinine (sCr) values at each considered time of serial assessment**.

**Figure 3:**
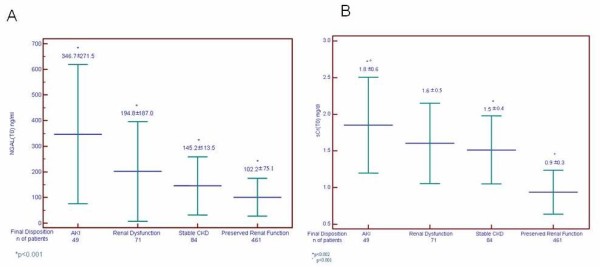
**Mean ± SD neutrophil gelatinase-associated lipocalin (NGAL) (A) and serum creatinine (sCr) (B) at T0 by final diagnosis**.

**Figure 4:**
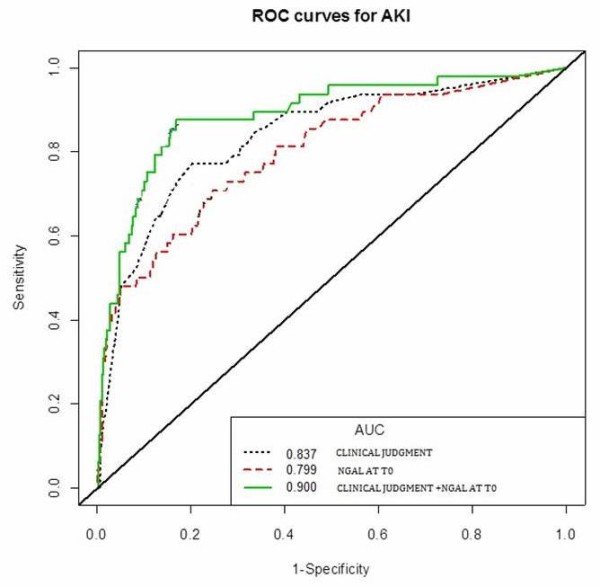
**T0 receiver-operating characteristic (ROC) curves by adjudicated acute kidney injury (AKI) based on neutrophil gelatinase-associated lipocalin (NGAL), clinical judgment of developing AKI, and NGAL combined with clinical judgment**.

The mean (± SD) blood NGAL and sCr levels in the whole study population, in AKI, and in NO AKI groups at each measured time are shown in Figure 2. NGAL mean values in AKI patients were significantly higher at each considered time point compared to patients with NO AKI (*P *< 0.001). sCr mean values were also higher in AKI patients in comparison to NO AKI patients at each considered time (*P *< 0.001). Table 2 lists NGAL values in the AKI and NO AKI groups based on discharge diagnosis. In patients with sepsis, pneumonia and acute decompensated heart failure (ADHF) NGAL values at T0 in AKI patients were significantly higher compared to patients with NO AKI (*P *< 0.001). Figure 3 shows the difference between NGAL (Figure 3A) and sCr (Figure 3B) levels at admission for patients with adjudicated AKI, renal dysfunction, stable CKD or preserved renal function. Between groups, NGAL values in adjudicated AKI patients were significantly higher compared to the other three groups of patients (Figure 3A). Admission mean sCr level in patients with adjudicated AKI was significantly increased compared to patients with stable CKD (*P *< 0.002) or preserved renal function (*P *< 0.001), while there was no difference between sCr levels at admission for patients with AKI compared to patients with renal dysfunction. The ROC curve analysis showed that NGAL at baseline alone or combined with the ED physician's initial clinical judgment was highly predictive of RIFLE AKI (Figure 4). There were no differences between AUC of NGAL (T0) (0.80 + 0.04) in comparison to AUC of eGFR (T0) obtained by MDRD (0.80 + 0.04), and to AUC of eGFR obtained by Cockroft-Gault (0.80 + 0.04). However, the AUC of NGAL plus the ED physician's clinical judgement was significantly higher than the AUC of eGFR (either obtained by MDRD or by Cockroft-Gault) plus the ED physician's clinical judgement (0.90 vs. 0.78, *P *= 0.022 and 0.90 vs. 0.78, *P *= 0.020, respectively). Serial assessment of T0 and T6 hours NGAL provides a high NPV (98%) in ruling out the diagnosis of AKI within 6 hours of patients' ED arrival. In an NRI analysis, the same model combining NGAL (T0) with the ED physician's clinical judgement was compared to a model combining admission sCr (T0) results with the ED physician's clinical judgement and the NRI (95% CI) was 32.4 percentage points (3.02 to 61.8), meaning that the percentage correct in the classification of AKI improved significantly by 32.4 points.

**Table 2:** Mean ± SD of NGAL (T0) values in AKI vs.

	T0 NGAL AKI GROUP (*n *= 49)	T0 NGAL NO AKI GROUP (*n *= 616)	*P *value
Sepsis (*n *= 51)	542.6 ± 298.7 (11)	213.7 ± 229.5 (40)	*P *< 0.001
Local infection (*n *= 121)	357 ± 242.8 (4)	171.1 ± 177 (117)	*P *< 0.04
Acute decompensated heart failure (*n *= 108)	216.2 ± 230 (10)	129.7 ± 83.2 (98)	*P *< 0.001
Stroke (*n *= 38)	114.5 ± 84.7 (4)	104.6 ± 76.8 (34)	*P *= 0.48
Severe dehydration (*n *= 9)	511.6 ± 124.5 (3)	261.8 ± 215.3 (6)	*P *< 0.001
Liver cirrhosis (*n *= 11)	362 ± 390.3 (2)	247.5 ± 298 (9)	*P *< 0.01
Decompensated diabetes (*n *= 10)	0	109 ± 60.2 (10)	

NO AKI group of patients on the basis of diagnosis at discharge.

n = number of patients. AKI, acute kidney injury; NGAL, neutrophil gelatinase-associated lipocalin; SD, standard deviation.

**Figure 5:**
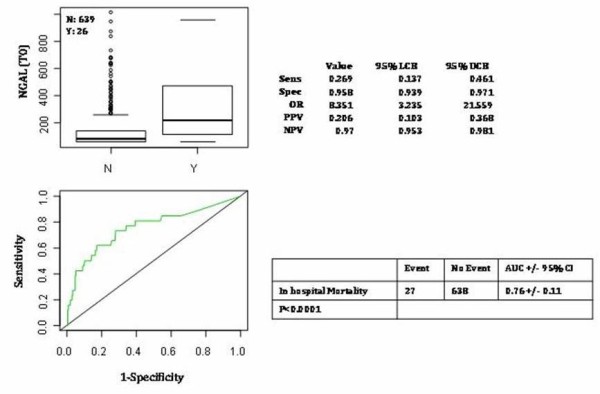
**Neutrophil gelatinase-associated lipocalin (NGAL) (T0) predictive value of in-hospital mortality**. Odds ratio (OR), negative predictive value (NPV), positive predictive value (PPV) on the basis of NGAL T0 threshold 400 ng/ml. N = no exitus, Y = yes, exitus.

Table 3 represents the AUCs, OR and the sensitivity, specificity, NPV, and PPV of NGAL thresholds 150≥ ng/ml and ≥400 ng/ml for the RIFLE diagnosis of AKI as well as the secondary definitions based on AKIN criteria, oliguria. In our data an NGAL cutoff >400 ng/ml gives the highest odds ratio (OR 22.5) for the prediction of the final RIFLE diagnosis of AKI. Total in-hospital mortality was 4.06%. Figure 5 demonstrates the relationship between admission NGAL at cutoff of 400 ng/ml and in-hospital mortality (OR 8.3, *P *< 0.001). In order to evaluate the additive value of NGAL and sCr in the risk stratification of patients for the outcomes of in-hospital mortality and the need for RRT, we applied the high specificity threshold for NGAL (400 ng/mL) and the threshold for sCr increase according to RIFLE 'R' criteria to assign patients to four groups.

**Table 3:** NGAL (T0) values on the basis of diagnosed AKI by RIFLE criteria, AKIN criteria (48 hrs sCr increase), oliguria with two NGAL thresholds (≥150 ng/ml and ≥400 ng/ml).

	Threshold ≥150 ng/ml (*n *= 160 patients: AKI *n *= 30/NO AKI *n *= 130)							Threshold ≥400 ng/ml (*n *= 34 patients: AKI *n *= 18/NO AKI *n *= 16)						
	**Sensitivity** **(95% CI)**	**Specificity** **(95% CI)**	**OR** **(95% CI)**	**PPV**	**NPV**	**AUC**	***P***	**Sensitivity** **(95% CI)**	**Specificity** **(95% CI)**	**OR** **(95% CI)**	**PPV**	**NPV**	**AUC**	***P***

**DIAGNOSIS OF AKI**														

RIFLE AKI	0.62 (0.48-0.74)	0.78 (0.75-0.82)	6.24 (3.37-11.55)	0.18	0.96	0.79	<0.001	0.37 (0.25-0.51)	0.97 (0.95-0.98)	22.53 (10.46-48-51)	0.52	0.95	0.79	<0.001

AKIN CRITERIA (48 hrs sCr increase)	0.70 (0.39-0.89)	0.77 (0.73-0.80)	7.82 (1.99-30.65)	0.04	0.99	0.84	<0.001	0.40 (0.16-0.68)	0.95 (0.94-0.97)	15.46 (4.10-58.30)	0.13	0.99	0.84	<0.001

OLIGURIA (<0.5 ML/kg/body weight in 6 hrs)	0.78 (0.52-0.92)	0.77 (0.73-0.80)	12.32 (3.39-44.75)	0.07	0.99	0.81	<0.001	0.42 (0.21-0.67)	0.95 (0.93-0.97)	16.42 (5.33-50.53)	0.17	0.98	0.81	<0.001

AKI, acute kidney injury; AKIN, Acute Kidney Injury Network; AUC, area under the curve; CI, confidence interval; ED, emergency department; EDTA, NGAL, neutrophil gelatinase-associated lipocalin; NPV, negative predictive value; OR, odds ratio; PPV, positive predictive value; RIFLE criteria, Risk, Injury, Failure, Loss, and End-Stage Kidney Disease criteria.

**Figure 6:**
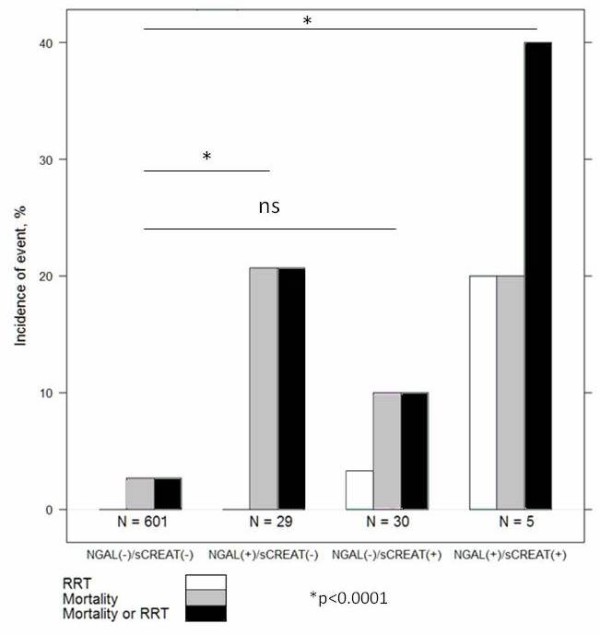
**Risk stratification comparing groups of patients obtained by combining T0 serum creatinine (sCr) +/- and neutrophil gelatinase-associated lipocalin (NGAL) +/-**. Rates of clinical events for the combined endpoint of renal replacement therapy (RRT) or in-hospital mortality.

We stratified subjects in sCr+ (sCr ≥50% increase) or sCr*_- _*(sCr <50% increase) and NGAL+ (NGAL >400 ng/ml) or NGAL*_- _*(NGAL <400 ng/ml). There were 2 patients in our cohort who required RRT and 26 who suffered in-hospital mortality. For the combined endpoint of RRT or mortality there were 27 patients. Figure 6 shows that the event rate for the combined endpoint of in-hospital mortality and the need for RRT were highest for patients in the sCr+ and NGAL+ group and lowest for patients who were in the sCr*_- _*and NGAL*_- _*group. Furthermore, the patients that were NGAL+ and sCr*_- _*had a higher incidence of the combined endpoint than those who were NGAL*_- _*and sCr+. The OR for the combined endpoint of RRT and in-hospital mortality for the NGAL+, sCr*_- _*group compared to the group with both biomarkers negative was 9.45 (2.77 to 28.57, *P *= 0.0002), while the NGAL*_-_*, sCr+ group had an OR of 4.05 (0.71 to 15.46, *P *= 0.056) and the group with both markers positive had an OR of 23.84 (1.87 to 223.43, *P *= 0.008).

## Discussion

The identification of AKI in patients presenting with heterogeneous comorbidities is difficult because sCr rise and decreasing urinary output take time to detect [29].

Our study demonstrated that blood NGAL measurements were useful in predicting development of AKI at time of admission from the ED. This result confirms data available from the literature on the same cohort of patients [4,5,13,29-33]. From our data, the ED physician's judgment lacked sensitivity and specificity, in predicting the RIFLE diagnosis in 27% of the AKI cohort, while missing 20% of those with a final diagnosis of AKI. The addition of NGAL to the ED physician's assessment not only decreased the number of patients erroneously suspected of AKI at initial presentation (false positives), but also identified additional patients that were missed in the early stages of this assessment.

From our results, for the diagnosis of AKI, it seems that there was no difference between the AUCs obtained with NGAL alone or with eGFR, calculated either with MDRD or Cockroft-Gault. This suggests that the ability of NGAL to diagnose AKI is not inferior to the current commonly used standard of care marker. However, when NGAL was added to the ED physician's clinical judgment the AUC was increased compared to clinical judgment alone or compared to the combination of clinical judgment and eGFR (MDRD or Cockroft-Gault). Moreover the advantage of coupling NGAL and the ED physician's clinical judgment is demonstrated by the increase of AKI prediction (NRI = 32.4%), these results imply that the addition of a blood NGAL could more accurately triage patients on arrival than the use of current standards alone.

The use in our study of a rapid point-of-care platform testing system that provides quantitative NGAL results from whole blood in 15 minutes [26] adds important clinical implications to our results since the time to diagnosis in the ED is inversely related to the optimal patient outcome. Inherent to this is prompt initiation of treatment and timely patient disposition. The use of NGAL as an early biomarker of kidney tubular injury could have important clinical implications in the ED setting; an early detection of kidney damage may lead to more timely specialist consultations, specific therapeutic approaches and final dispositions. Consequently, this may lead to a decrease in the progression of kidney damage with improved patient outcomes and reduced health care expense [34]. This is also confirmed from our data which demonstrate that the patients presenting to the ED with NGAL and sCr positive values are at higher risk to develop in-hospital events such as death and need of RRT. For patients with NGAL and sCr within normal ranges, the ED physician could promptly rule out AKI while in subjects with elevated NGAL and/or sCr values he should probably consider specific therapeutic options. To the best of our knowledge, this is the first multicenter study performed on the blood samples of this biomarker applied to all patients hospitalized from the ED [4,5]. In fact the recent paper from Nickolas *et al. *[4] was also an ED multicenter study but using urinary NGAL. The incidence of AKI (7%) in our cohort of patients was similar to that obtained by Nickolas *et al. *[5]. While Shapiro *et al*. also investigated the use of a blood NGAL POCT assessment in the ED, they considered only patients with suspected sepsis [22]. Our cohort included patients with sepsis, ADHF, pneumonia, stroke, severe dehydration, liver cirrhosis and several other critical conditions, many of which are often contemporaneously present, especially in older patients. Consequently our data are applicable to a large number of undifferentiated patients coming to the ED requiring hospitalization. Our results are more generalizable to the undifferentiated ED population because the recently published data on the role of blood NGAL in detecting AKI in the ED have been showed in restricted populations such as: patients with sepsis [22], or with lower respiratory tract infection, or with cardiorenal syndrome by using a multi-marker approach [35-44].

In our study, the incidence of final expert-adjudicated AKI was higher than AKI as defined by RIFLE criteria, AKIN criteria or the development of oliguria during hospitalization [8,9]. This result raises the possibility that RIFLE or AKIN criteria [8,9], currently suggested as the standard methods for diagnosing AKI, may have limited utility in the early detection of AKI in the ED. This is consistent with the findings of Haase *et al*. and Nickolas *et al. *[4,13]. Nevertheless, since in the study a pre-study sCr level was not available, we cannot exclude (as a limitation of the results) that a significant proportion of patients could have already had an increase in sCr and NGAL values before ED presentation.

In the last decade, data have been published on a single measurement of NGAL with a wide range of specificity and sensitivity for diagnosis of AKI [5,16,20,37-39]. This wide variability could be explained by differing times of measurement and different NGAL cutoffs that have been proposed for the diagnosis of AKI. We decided to use the cutoff of 150 ng/ml because it is internationally considered a high sensitivity threshold for AKI prediction [20,22,40]. A cutoff of 400 ng/ml has been demonstrated to be a high specificity threshold based upon international analysis that has been done on other data sets [21]. For septic patients, other authors considered NGAL cutoff values that ranged from 150 to 400 ng/ml [22,41]. Still others have used a cutoff value of 170 ng/ml, demonstrating that NGAL predicts 48 to 72 hour development of type 1 cardiorenal syndrome with NPV of 100% and PPV of 50% [18,38,42,43]. Building a ROC curve on the basis of NGAL T0, we obtained a cutoff value of 137 ng/ml for final diagnosis of AKI. This is very similar to the 150 ng/ml expressed in the literature [20].

Using a cutoff of 150 ng/ml and two serial NGAL measurements (T0, T6), we could establish a NPV of 98% within 6 hours of the patient's arrival. In the acute setting, the possibility of ruling out the occurrence of severe diseases such as AKI is of great importance [38,39,43,44]. On the other hand, NGAL value above cutoff of 400 ng/ml seems to be very specific for ruling on the diagnosis of AKI. Consequently, it appears that with NGAL, similar to the use of B-type natriuretic peptide (BNP) for diagnosis of heart failure [38] and for many other biomarkers, there could exist a grey zone where the clinical judgment coupled with biomarker assessment is crucial to the prompt and accurate diagnosis of AKI [45]. In our study, blood NGAL also demonstrated the ability to differentiate intrinsic AKI from other renal diseases/dysfunction at initial assessment. NGAL values were significantly higher in patients with AKI when compared to patients with renal dysfunction or stable CKD. These results are similar to the Nickolas studies on urinary NGAL [4]. From our results, blood NGAL value in this group of patients with renal dysfunction was significantly higher compared to patients with stable CKD and to patients with preserved renal function, a finding that is consistent with those of Singer *et al .*[38]. This allows clinicians to distinguish between chronic disease and early reversible kidney damage. From our results it was evident that sCr was not able to distinguish this difference, since the value of sCr in this group with renal dysfunction was not increased at baseline evaluation compared to patients with stable CKD or with preserved renal function. From our data, NGAL could not only be considered a diagnostic marker but also a prognostic biomarker. Admission NGAL has been shown to be able to predict in-hospital mortality with a high OR (8.3) when a threshold of 400 ng/ml was used. Admission NGAL value above 400 ng/ml had a high AUC (0.76 +/- 0.11) for in-hospital mortality. The ability to predict which patients will likely need RRT and which have a high probability of in-hospital death has understated importance in resource utilization.

## Conclusions

In summary, our study demonstrated that admission blood NGAL measurements are useful in the early diagnosis of AKI. Baseline NGAL measurement allows detection of AKI earlier than sCr. Improved diagnostic performance was demonstrated when NGAL was combined with clinical judgment. NGAL is also able to distinguish intrinsic AKI from early reversible kidney dysfunction while sCr cannot.

Blood NGAL levels detected development of acute renal injury at 6 hours, nearly 2 days earlier than sCr increases at 48 hours. Blood NGAL assessment at the moment of hospital admission from the ED predicted the combined outcome of RRT and in-hospital mortality.

The use of this kidney injury biomarker in the early detection of renal involvement and risk stratification of patients at the time of ED presentation may permit earlier interventions and more appropriate patient management strategies.

## Key messages

• Admission blood NGAL measurements are useful in the early diagnosis of AKI.

• Baseline NGAL measurement allows detection of AKI earlier than sCr.

• Blood NGAL assessment at the moment of hospital admission from ED predicted the combined outcome of RRT and in-hospital mortality.

## Abbreviations

ADHF: acute decompensated heart failure; AKI: acute kidney injury; AKIN: Acute Kidney Injury Network; AUC: area under the curve; BMI: body mass index; BNP: B-type natriuretic peptide; CKD: chronic kidney disease; CI: confidence interval; CT: computed tomography; ED: emergency department; EDTA: ethylenediaminetetraacetic Acid; eGFR: estimated glomerular filtration rate; ICU: intensive care unit; IQR: interquartile range; KDIGO: Kidney Disease: Improving Global Outcomes; KDOQI: Kidney Disease Outcome Quality Initiative; MDRD: modification of diet in renal disease; NGAL: neutrophil gelatinase-associated lipocalin; NPV: negative predictive value; NRI: net reclassification index; OR: odds ratio; POCT: point-of-care test; PPV: positive predictive value; RIFLE criteria: Risk: Injury: Failure: Loss: and End-Stage Kidney Disease criteria; ROC: receiver-operating characteristic; RRT: renal replacement therapy; sCr: serum creatinine; SD: standard deviation.

## Competing interests

Prof. Di Somma received a grant for the development of this study and he is consultant for Alere. The other authors declare that they have no competing interests.

## Authors' contributions

SDS contributed to the conception and design of the study, to the interpretation of data, drafting the article and to the final approval of the version to be published. PM and GC contributed to the conception and design of the study. LM and BDB contributed to the interpretation and analysis of data, drafting the article and revising it, and to the final approval of the version to be published. RM, EF, PB, PN and BG contributed to the acquisition, interpretation and analysis of data. LP contributed to revising it critically for intellectual content and to the final approval to the version to be published. EDS contributed to the statistical analysis and to drafting the article. All authors have read and approved the manuscript for publication.
